# Oesophago-Cutaneous Fistula Due to a Broken Titanium Plate of Anterior Cervical Plate Fixation

**Published:** 2011-06-01

**Authors:** N N Kawoosa, A R Bhat, B R Zargar

**Affiliations:** 1 Department of Neurosurgery, Sher-i-Kashmir Institute of Medical Sciences (SKIMS), Soura, Srinagar, Kashmir, 190011, India; 2 Senior Resident, Sher-i-Kashmir Institute of Medical Sciences (SKIMS), Soura, Srinagar, Kashmir, 190011, India

**Keywords:** Anterior cervical plate, fixation, Cervical spine, Complication, Esophago-cutaneous fistula

Dear Editor,

Anterior cervical spine fusion and plate fixation is a valuable procedure for the treatment of cervical spinal injury, spondylitic disease and intervertebral disc diseases.[1] Esophageal perforation is one of the rare but serious complications following anterior cervical spinal surgery. Esophageal injury is usually due to the surgical exposure or the foreign metallic implant which usually manifests in the early post operative period. Potential risk factors include revision spine surgery, difficult surgical exposures, surgery for spine trauma, and a prior history of esophageal disease or surgery. Esophageal injury due to a broken metallic implant is very rare. A forty-six-year-old man who was involved in an accident underwent a lower partial corpectomy of the seventh cervical vertebral body, C7 D1 dissectomy through anterolateral approach, followed by four whole titanium plate fixation (compressive plate) with autologous bone graft. On the eighth postoperative day, he developed high grade fever, complained

of dysphagia, odynophagia and persistent foreign body sensation in the throat. Subsequently, ingested liquids and thick pus started oozing from the suture line in the neck. Computed tomography (CT) scan showed the titanium plate broken into two pieces, the broken fragment eroding the esophageal wall. The patient underwent emergency reexploration and the broken plate was removed and replaced by a new one. How did the titanium plate broke is still a mystery as it is very hard to believe that titanium plate can break into two pieces. Inappropriate bender used for bending the plate before surgery is the only answer we can think of.

The diagnosis of such an esophageal injury remains difficult; patient symptoms are nonspecific, and, diagnostic imaging is limited. Dysphagia, especially associated with odynophagia following anterior cervical spine surgery should be evaluated for graft/implant-related complications.[2] The presence of air in the fascial planes of the neck and increasing soft tissue swelling in the postoperative X-ray suggest esophageal perforation.2 The diagnosis of esophageal perforation can be confirmed by contrast esophagography with a high accuracy rate.[3][4] CT scan helps in delineating the location and condition of the implant,extent of an underlying abscess and possible extension of the abscess along the prevertebral space.[5] Early surgical intervention and removal of the broken fragment is the treatment of choice. Early detection and treatment may reduce the complications associated with injury, including patient death.
